# Ginkgolic Acid Inhibits Herpes Simplex Virus Type 1 Skin Infection and Prevents Zosteriform Spread in Mice

**DOI:** 10.3390/v13010086

**Published:** 2021-01-09

**Authors:** Maimoona S. Bhutta, Oren Shechter, Elisa S. Gallo, Stephen D. Martin, Esther Jones, Gustavo F. Doncel, Ronen Borenstein

**Affiliations:** 1 Department of Microbiology and Molecular Cell Biology, Eastern Virginia Medical School, Norfolk, VA 23507, USA; bhuttam@evms.edu (M.S.B.); orenshechter@yahoo.com (O.S.); MartinS1@EVMS.EDU (S.D.M.); JonesEB@EVMS.EDU (E.J.); 2 Board-Certified Dermatologist and Independent Researcher, Norfolk, VA 23507, USA; esgallomd@hotmail.com; 3 CONRAD, Arlington, VA 22209, USA; DoncelGF@EVMS.EDU; 4 Department of Obstetrics and Gynecology, Eastern Virginia Medical School, Norfolk, VA 23507, USA

**Keywords:** ginkgolic acid, antiviral, herpes simplex type 1, acyclovir-resistance, zosteriform infection, fusion inhibition, virucidal activity

## Abstract

Herpes simplex virus type 1 (HSV-1) causes a lifelong latent infection with an estimated global prevalence of 66%. Primary and recurrent HSV infections are characterized by a tingling sensation, followed by an eruption of vesicles, which can cause painful erosions. Commonly used antiviral drugs against HSV infection are nucleoside analogues including acyclovir (ACV), famciclovir, and valacyclovir. Although these nucleoside analogues reduce morbidity and mortality in immunocompetent individuals, ACV-resistant HSV strains (ACV^R^-HSV) have been isolated from immunocompromised patients. Thus, ACV^R^-HSV infection poses a critical emerging public health concern. Recently, we reported that ginkgolic acid (GA) inhibits HSV-1 by disrupting viral structure, blocking fusion, and inhibiting viral protein synthesis. Additionally, we showed GA affords a broad spectrum of fusion inhibition of all three classes of fusion proteins, including those of HIV, Ebola, influenza A and Epstein Barr viruses. Here we report GA’s antiviral activity against HSV-1 skin infection in BALB/cJ mice. GA-treated mice demonstrated a significantly reduced mortality rate and decreased infection scores compared to controls treated with dimethylsulfoxide (DMSO)-vehicle. Furthermore, GA efficiently inhibited ACV^R^-HSV-1 strain 17+ in vitro and in vivo. Since GA’s mechanism of action includes virucidal activity and fusion inhibition, it is expected to work alone or synergistically with other anti-viral drugs, and we anticipate it to be effective against additional cutaneous and potentially systemic viral infections.

## 1. Introduction

Herpes simplex virus type 1 (HSV-1) is a prevalent human pathogen with an estimated global prevalence of 66% (3752 million people) [1,2]. HSV-1 causes a lifelong latent infection, and it is primarily transmitted through fluids or droplets from cutaneous or mucosal lesions [2,3]. HSV-1 invades its human host after contact with irritated skin or mucosal epithelium leading to infectious viral shedding, tissue damage, and formation of vesicles and erosions [4,5]. Following primary infection, the virus enters the sensory nerve endings and neural ganglia, where it establishes a lifelong latency, and with reactivation, recurs at the mucosal and cutaneous sites.

The primary mechanism of viral entry occurs through the fusion of the viral envelope with the host cell’s plasma membrane, which is followed by transport of the viral capsid to the nucleus [6]. Regulation of HSV-1 transcription occurs in sequential three phases of gene expression, beginning with the production of immediate-early (IE) or α-genes, early (E) or β-genes, and late (L) or γ-genes. α/IE genes, which include infected cell polypeptides (ICP), ICP0, ICP4, ICP22, and ICP27, regulate early gene expression of viral replication. The transcription of α/IE genes, requiring the presence of viral trans-activator VP16 in vivo, can also occur in the absence of prior or de-novo viral protein synthesis [6,7,8,9]. The primary function of α/IE gene-encoded proteins is the activation of β/E gene expression. β/E gene proteins, such as thymidine kinase (TK, UL23) and HSV DNA polymerase (pol, UL30), are principally involved in viral genome replication, suppression of α/IE genes, and the activation of γ/L genes [6,9]. γ/L genes primarily encode structural proteins, leading to the assembly and release of infectious particles [6,9].

Most HSV infections begin with a tingling sensation before the appearance of vesicles, causing unsightly and sometimes painful erosions. The lesions typically heal within one week, though they may take up to 3–4 weeks to resolve depending on the individual; immunocompromised individuals have a longer route to recovery. Following primary infection, the virus enters the sensory nerve endings and neural ganglia, where it establishes a lifelong latency, and with reactivation, recurs at the mucosal and cutaneous sites. HSV-1 establishes latency in the host sensory ganglia and central nervous system (CNS) that innervate the site of initial infection [2,3,8,9]. During latency, viral transcription in the ganglia is limited to latency-associated transcripts (LATs). HSV-1 LATs have been reported to limit α/IE gene expression in vitro and limit the accumulation of lytic gene transcripts during acute and latent infection in murine models [10]. Reactivation of virus can lead to asymptomatic or symptomatic shedding of HSV. Asymptomatic shedding is often defined as detection of HSV in the absence of lesions, or if symptoms are present, they are not recognized by the patient or clinician as related to HSV [7]. Symptomatic shedding often results in recurrent disease at the initial infection site, resulting in gingivostomatitis, orolabial herpes, herpes stromal keratitis and fatal herpes sporadic encephalitis (HSE) [2,3,6]. In immunocompromised patients and neonates, HSV-1 can disseminate to the brain, lungs, and or liver with potentially fatal outcomes [11].

The most common class of antiviral and prophylaxis drugs used to treat HSV infection are nucleoside analogues such as acyclovir (ACV), penciclovir, famciclovir, and valacyclovir [6]. ACV is a guanosine analog that must be phosphorylated by viral thymidine kinase (UL23) and then by cellular kinases into its active triphosphate form. The active form of ACV acts as a competitive inhibitor of viral DNA polymerase, and it is incorporated into the nascent viral DNA to block further viral replication [6,12,13]. Although improvements in treatments have dramatically reduced morbidity and mortality in immunocompetent individuals, HSV clinical isolates with reduced susceptibility or resistance to ACV have been increasingly isolated from immunocompromised patients, with a prevalence of 14% and 36% from stem cell transplant recipients [14,15,16]. Viral mutations resulting in ACV-resistance have been found in both TK UL23 (accounting for 95% of clinical isolates) and pol UL30 gene (5% of clinical isolates) [11,13]. Mechanisms of resistance are related to mutations in UL23, such as nucleotide insertions, deletions, or substitutions within the guanosine or cytidine homopolymer repeats. These mutations cause a frameshift, which results in the synthesis of non-functional/truncated TK [12,14]. Thus, ACV^R^-HSV infection remains a critical problem for many patients, especially those with HIV infection [4,16,17]. The emergence of resistant viral strains generates a genuine need to find and develop new anti-HSV agents.

Ginkgolic acid (GA), a mixture of 2-hydroxy-6-alkylbenzoic acids, was extracted from the leaves and seed coats of *Ginkgo biloba*, which have been used as herbal supplements in Chinese medicine since the 16th century [18,19]. Previous research has found GA to have beneficial effects including antitumor [20,21], anti-HIV [22], anti-bacterial [23], anti-inflammatory [24], and molluscicidal properties [25]. GA has also shown the potential to rescue amyloid-β (Aβ)-induced synaptic impairment [26]. Various mechanisms of action of GA have been postulated, including impairment of the formation of the E1-SUMO intermediate by directly binding to E1, thus inhibiting SUMOylation [27], inhibition of non-specific SIRT [28], inhibition of FAS and micromolar range cytotoxic activities against human cancer cells [29], and activation of protein phosphatase type-2 C [30].

Recently, we reported that GA inhibits HSV-1 by inhibition of both fusion and viral protein synthesis. Additionally, we showed a broad spectrum of fusion inhibition by GA of all three classes of fusion proteins, including those seen in human immunodeficiency virus (HIV), Ebola virus (EBOV), influenza A virus (IAV), and Epstein Barr virus (EBV). Our experiments suggest that GA inhibits virion entry by blocking the initial fusion event and by its virucidal activity [31].

Since GA has potent antiviral activity, we hypothesized that it would effectively treat topically active HSV-1 lesions. In this paper, we report for the first time that GA has antiviral activity against HSV-1 skin infection in BALB/cJ mice. GA formulated in 2.5% hydroxyethylcellulose (HEC) gel or polyethylene glycol (PEG) resulted in a significant reduction in mortality and infection scores compared to DMSO-treated vehicle control. Furthermore, GA efficiently inhibited the acyclovir-resistant strain of HSV-1 in vitro and in vivo against HSV-1 cutaneous infection.

## 2. Materials and Methods

### 2.1. Animals and Ethics Statement

All studies were performed in BALB/cJ female mice (5–6 weeks of age; 15–20 g weight) purchased from Jackson Laboratory (Bar Harbor, ME, USA). The animals were single-housed in sterile microisolator cages, kept on a 12:12 light-dark cycle, and maintained within the biosafety level 2 animal facility. All procedures were performed under a protocol approved by Eastern Virginia Medical School’s Institutional Animal Care and Use Committee (Protocol#18-012) following the National Institute of Health’s Guide for the Care and Use of Laboratory Animals.

### 2.2. Cells and Virus

Vero cells, an African green Monkey kidney epithelial cell line (ATCC^®^ CCL-81™), were used to grow and titer viral stock. Vero cells were propagated in Dulbecco’s Modified Eagle Medium (DMEM; Cat# sc-224478, Ultra-Cruz, Dallas, TX, USA) supplemented with 5% heat-inactivated fetal bovine serum (HI FBS; Cat# 10082-147, Gibco, Waltham, MA, USA) and 1% penicillin and streptomycin (P/S; Cat# 15140-122, Gibco), referred to as DMEM/5% FBS also mentioned as growth media. GFP-HSV-1 strain 17+ was a generous gift from Dr. Peter O’Hare [32]. Viral infections in in vitro experiments were carried out in medium 199 (1 X) (Cat# 11150-059, Gibco), supplemented with 1% HI FBS (Cat# 10082-147, Gibco) and 1% P/S (Cat# 15140-122, Gibco), referred to as 199/1% FBS.

### 2.3. Generation of an ACV^R^-GFP-HSV-1 Strain 17+

**Figure 1:**
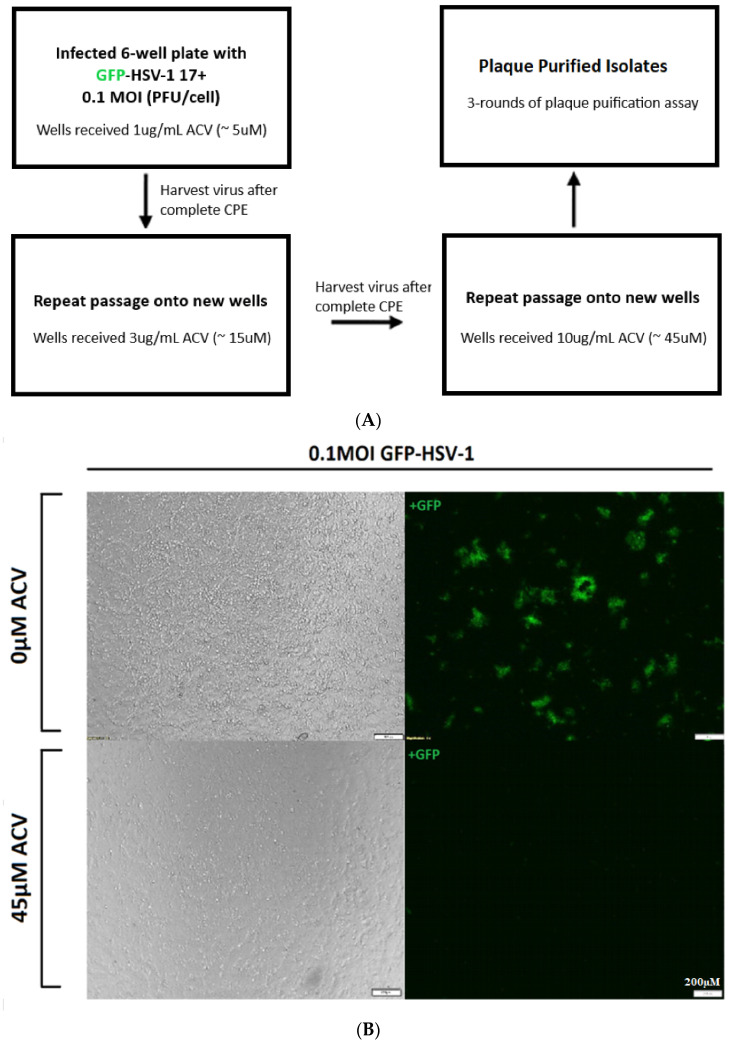

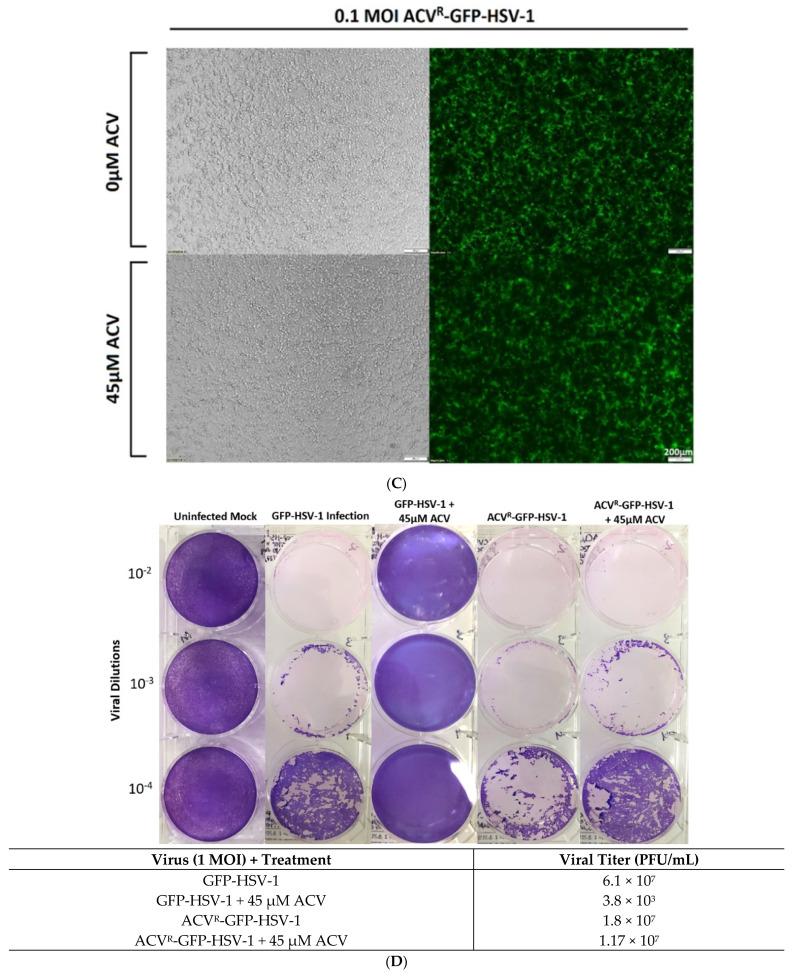

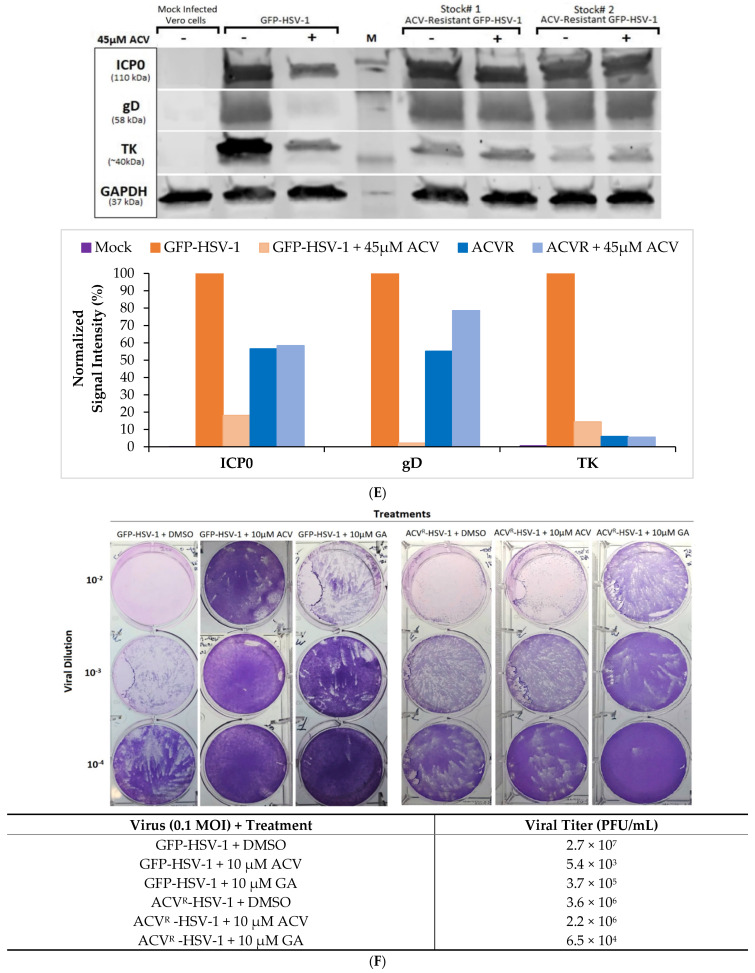
Verification of acyclovir-resistant (ACV^R^) HSV-1 infection in Vero cells. (**A**) Flow chart of the methodology used to create ACV-resistant (ACV^R^) mutants, adapted from Sarisky et al. [33]. Verification of acyclovir-resistant (ACV^R^)-GFP-HSV-1 17+ in Vero cells. (**B**) Fluorescent images of 0.1 MOI GFP-HSV-1 infection in Vero cells with or without 45 µM ACV. FITC filter with excitation of 490 nm and emission of 525 nm was used to visualize viral plaques at 40× magnification; Scale bar represents 200 µm. (**C**) Fluorescent images of 0.1 MOI ACV^R^-GFP-HSV-1 infection in Vero cells with or without 45 µM ACV. FITC filter with excitation of 490 nm and emission of 525 nm was used to visualize viral plaques at 40× magnification. Scale bar represents 200 µm. (**D**) Plaque Assays of viral stocks (1 MOI) with or without 45 µM ACV in Vero cells in viral dilutions of 10^−2^ to 10^−4^, shown as qualitative image and in a table with averaged viral titers. (**E**) Western Blot analysis using antibodies directed against HSV-1 infected cellular protein 0 (ICP0; Santa-Cruz, sc-53070), glycoprotein D (gD; Santa-Cruz, sc-69802), thymidine kinase (TK; Invitrogen, PA5-67984), and GAPDH (0411; Santa-Cruz, sc-47724). M: Chameleon 800 Pre-stained Protein Ladder (Li-Cor, 928-80000). Signaling intensities of bands for each antibody have been normalized (as a percentage) to GFP-HSV-1. ACVR: ACV^R^-HSV-1. (**F**) Plaque Assays of GFP-HSV-1 17+ (0.1 MOI) and ACV^R^- HSV-1 (0.1 MOI) with various treatments in Vero cells such as DMSO (1:2000), 10 µM GA, and 10 µM ACV in viral dilutions of 10^−2^ to 10^−4^, shown as qualitative image and in a table with averaged viral titers.

Previous literature has demonstrated that HSV isolates from cell culture are composed of a heterogeneous population, containing pre-existing antiviral-resistant thymidine kinase (TK) variants. Therefore, exposure of nucleoside analogues, such as acyclovir, in cell culture can provide selective pressure that can lead to enrichment of drug-resistant TK variants [33]. GFP-HSV-1 was used to generate an acyclovir-resistant (ACV^R^) mutant strain as previously described by Sarisky et al. (Figure 1A) with the following changes: 70–80% confluent Vero cells in a 6-well plate were infected with 0.1 MOI of GFP-HSV-1 in the presence of 1 µg/mL ACV (Cat#2513, Tocris, Minneapolis, MN, USA). After observing a complete cytopathic effect (CPE), the virus was harvested and passaged onto fresh Vero cells (6-well plate) in the presence of 3 µg/mL ACV. The virus was harvested again after complete CPE and passaged for a third time in Vero cells with 10 µg/mL ACV. Plaques from the third passage were isolated, and the viral stock underwent three rounds of plaque purification in T:25 flasks. Fluorescence microscopy (Model IX73, Olympus, Tokyo, Japan) was used to image ACV^R^-HSV-1 using the FITC filter (excitation: 490 nm and emission: 525 nm).

### 2.4. Plaque Assay Technique and Measuring Inhibition with Plaque Assay

The virus titer of GFP-HSV-1 (6.0 × 10^7^ PFU/mL) and ACV^R^-GFP-HSV-1 (1.8 × 10^7^ PFU/mL) was determined by standard plaque assays, as previously described by our lab [31]. The rescue activity of GA, following GFP-HSV-1 and ACV^R^-GFP-HSV-1 infection, was assessed in in vitro cell culture before in vivo experiments. The resistant activity of ACV^R^-GFP-HSV-1 was evaluated in cell culture in the presence of ACV. The Vero cell monolayer in a 6-well plate was incubated with DMEM/5% FBS containing 45 µM ACV for 1 h. The treatment was removed, and cells were incubated with 1 MOI of ACV^R^-GFP-HSV-1 or GFP-HSV-1 in 199/1% FBS media, for 1 h at 37 °C. After incubation, viral media was replaced with growth media containing 45 µM ACV for 20 h. The infected culture was collected, and the viral titer was measured using plaque assays, as previously described by Borenstein et al. [31]. To test the inhibitory activity of GA following ACV^R^-GFP-HSV-1 infection, 70–80% confluent Vero cells in a 6-well plate were pre-treated with 1:2000 dilution DMSO, 10 µM GA, or 10 µM ACV for 1 h at 37 °C. The treatment was removed, and the cells were incubated with 0.1 MOI of GFP-HSV1 or ACV^R^-HSV-1 in 199/1% FBS for 1 h at 37 °C. The viral media was then replaced with growth media containing the respective treatments for 20 h. The infected culture was collected, and the viral titer was measured using plaque assays [29].

### 2.5. Western Blot Analysis

Western blot analysis was conducted as previously described [31]. Infected cells were harvested and collected using low centrifugation (3000 rpm for 5 min). The cell pellet was lysed using RIPA buffer, protease inhibitor, and EDTA solution, as per manufacture instructions. Approximately 50 μg of protein per sample was subjected to further analysis. Proteins were electrophoretically separated on pre-cast 4-to-15% Mini-Protean TGX Gels (Cat#456-1084, Bio-Rad, Hercules, CA, USA) and transferred to 0.2 μm nitrocellulose membrane, Trans-Blot Turbo Transfer pack (Cat#1704158, Bio-Rad). The membrane was blocked by Odyssey^®^ Blocking Buffer (PBS; Cat#927-40000, Li-Cor, Lincoln, NE, USA) for 20 min before applying primary antibodies overnight at 4 °C. The mouse monoclonal antibodies, HSV-1 ICP0 (Cat# sc-53070, Santa Cruz, Dallas, TX, USA), HSV-1/2 gD (H170; Cat# sc-69802, Santa Cruz), and GAPDH (041; Cat# sc-47724, Santa Cruz) were used at a dilution of 1:200. Membranes were washed four times with TPBS and then reacted with the appropriate goat anti-mouse secondary antibody at a dilution of 1:10,000, according to the manufacturer’s instructions. The rabbit polyclonal antibody to thymidine kinase, HHV-1 TK (Cat#PA5-67984, Invitrogen, Carlsbad, CA, USA), was used at a dilution of 1:500, after stripping the membrane using NewBlot IR Stripping Buffer (Cat# 928-40028, Li-Cor). The membrane was washed four times with TPBS and then reacted with the appropriate IRDye donkey anti-rabbit secondary antibody at a dilution of 1:10,000, according to the manufacturer’s instructions. The Western Blot membrane was analyzed using Odyssey CLx (Li-Cor) and Image analysis software.

### 2.6. Skin Irritation Analysis

To assess whether the dermal application of GA poses irritation or signs of an allergic reaction, we used a modified skin irritation protocol based on Sekizawa et al. [34]. We used four female BALB/cJ mice per group, for a total of five groups. Under anesthesia, the hair on the right flank was clipped (1 × 2 cm^2^ area) and depilated with Nair (Nair™, Ewing, NJ, USA). Various concentrations of treatments [10 mM GA, 3 mM GA, 1 mM GA, 0 mM GA (DMSO only), or vehicle-treated control (hydroxyethyl cellulose gel only)] formulated in 2.5% hydroxyethyl cellulose (HEC) gel were applied on shaven skin using a sterile plastic end of a cotton swab. 2.5% HEC gel was provided by Dr. Gustavo Doncel (EVMS, Norfolk, VA, USA). Mice were caged separately to prevent grooming and licking of gel solutions of other cage mates. All animals were monitored daily post-gel administration for 10 days to observe skin irritation/redness or infection. Skin reactions were graded using the Organization for Economic Co-operation and Development (OECD) rating system [35,36].

### 2.7. Zosteriform Infection Model

Cutaneous HSV-1 infection experiments were conducted using the epidermal scarification-zosteriform model, as previously described by Goel et al. [37]. Unless otherwise stated, 5-to-6 week aged female BALB/cJ mice (N = 5 to 10/treatment) were anesthetized with gas inhalation isoflurane (4–5% ± 0.8–1 L/min for induction and 1–2% ± 0.8–1 L/min for maintenance) using a nose cone. Under anesthesia, the right flank skin, dorsal to the spleen’s posterior tip, was shaven (2 cm ± 0.5 cm), and the mice were chemically denuded using Nair cream. After 24 h, BALB/cJ mice were anesthetized with ketamine (120 mg/kg) and xylazine (12 mg/kg), and the shaved areas were scratched with the pointed-side of a 26-gauge needle. The scratches were shallow to avoid bleeding but encouraged viral penetration into the subcutaneous layer. Each mouse was inoculated with 6 × 10^4^ PFU of GFP-HSV-1 strain 17+ or 2.0 × 10^5^ PFU of ACV^R^-HSV-1.

### 2.8. Treatments

The inoculation sites on the mice (N = 10/treatment group) were treated with 10% DMSO (vehicle-control) formulated in 2.5% HEC gel, 10 mM GA in 2.5% HEC gel, or acyclovir (ACV) ointment, USP 5% (Amneal, Bridgewater, NJ, USA; control for the standard of care). We also evaluated the protective effects of GA formulated in an FDA-approved vehicle agent, polyethylene glycol (PEG; average M_n_ = 600; #202401-250 G, Sigma-Aldrich, St. Louis, MO, USA). Infected animals (N = 5 to 10/treatment group) were treated at the inoculation sites with either 10% DMSO (vehicle-control) formulated in PEG, 10 mM GA in PEG, or 10 mM ACV in PEG.

Each treatment was administered 1-to-2 h post-infection and continued twice daily (at 12-h intervals) for approximately 14 days. All solutions were applied using the plastic end of a sterile cotton swab. Disease at the inoculation site was scored by the appearance of vesicles & lesions, using the infection. Animals were monitored twice daily for 14 days post-infection for any signs of deterioration, as outlined in the currently approved protocol. Signs of disease at the inoculation site were scored and recorded by the appearance of vesicles and erosions. The outcomes were graded as follows: 0—no lesions; 1, 2—local site lesions; 3, 4, 5—distant site zosteriform lesions along the dermatome; with 6—progression to severely compromised health, and 7—death (Figure S1).

### 2.9. Statistical Analyses

For GFP-HSV-1 zosteriform infection studies, sample size (N = 10) and power (1-β = 85%) were calculated based on findings of previous studies using Gigacalculator [38]. For ACV^R^-HSV infection studies, the sample size of 5 was calculated based on the findings of zosteriform experiments of GFP-HSV-1 in BALB/cJ mice. The sample size (N = 5) and power (85%) of ACV^R^-HSV in vivo infection studies were calculated using Gigacalculator. Changes in survivability (i.e., time of death and final mortality) were analyzed by Kaplan-Meier analysis and Log-rank (Mantel-Cox) test in GraphPad Prism (version 9.0.0 for Windows, GraphPad Software, San Diego, CA, USA). Changes in infectivity scores were analyzed across each treatment using Student independent *t*-tests (2-tailed) and or 1-way ANOVAs, where appropriate, using GraphPad Prism.

## 3. Results

### 3.1. GA Inhibits Infection of HSV-1 & ACV^R^-HSV-1 In Vitro

We have previously reported GA’s in vitro inhibitory effect on HSV-1 infection [31]. Here we examined and verified the inhibitory effect of GA on ACV-resistant (ACV^R^) HSV-1 17+, generated in our lab.

Previous literature has demonstrated that HSV isolates from cell culture are composed of a heterogeneous population, containing pre-existing antiviral-resistant thymidine kinase (TK) variants. Therefore, exposure of nucleoside analogues such as ACV in cell culture can provide selective pressure that can lead to enrichment of drug-resistant TK variants [33]. ACV^R^ mutants were created, as previously described by Sarisky et al. [33]. Vero cells were infected with 0.1 MOI of GFP-HSV-1 in the presence of 5 µM ACV (1 µg/mL). The virus was harvested after a complete cytopathic effect (CPE) was observed. The harvested virus was passaged onto new Vero cells in the presence of an increasing concentration of ACV, 15 µM (3 µg/mL). The virus was harvested again after complete CPE was observed, and the passage was repeated in Vero cells with 45 µM ACV (10 µg/mL). Plaques from the third passage were isolated, and the viral stock underwent three rounds of plaque purification (See Figure 1A).

The resistant activity of ACV^R^-HSV-1 was assessed in cell culture in the presence of ACV. We pre-treated Vero cells with or without 45 µM ACV for 1 h at 37 °C. The inoculum was removed, and cells were infected for 1 h with 0.1 MOI of ACV^R^-HSV-1 per well. After incubation, viral media was replaced with growth media containing 45 µM ACV for 20 h. The infected cells and culture media were collected, and the viral titer was measured by plaque assays. To visually analyze acyclovir resistance, a fluorescence microscope was used to image 0.1 MOI of ACV^R^-HSV-1 with or without 45 µM ACV. Our results demonstrated that administration of 45 µM ACV in GFP-HSV-1 infected Vero cells substantially decreases the presence of infected cells (Figure 1B). In contrast, Vero cells infected with 0.1 MOI ACV^R^- HSV-1 retain the level of infectivity in the presence and absence of 45 µM ACV (Figure 1C). The images of the plaque assays demonstrate qualitative effects whereby the administration of 45 µM ACV in GFP-HSV-1 infected Vero cells substantially decreased the presence of infected cells. In contrast, Vero cells infected with ACV^R^-HSV-1 retained the level of infectivity in the presence and absence of 45 µM ACV (Figure 1D). Likewise, our plaque assay showed an approximately 4-log decrease (from 1.5 × 10^7^ PFU to 3.8 × 10^3^ PFU) in viral titer between GFP-HSV-1 infected cells treated with 45 µM ACV compared to their control of infected cells. In contrast, we did not observe a decrease in the viral titer of ACV^R^-GFP-HSV-1 with or without 45 µM ACV, with viral titers of 1.2 × 10^7^ PFU and 1.17 × 10^7^ PFU, respectively.

Previous studies have shown that clinical isolates of ACV^R^-HSV infection express low-levels of TK activity [39]. Three different phenotypes of thymidine kinase mutants have been identified in ACV^R^-HSV isolates: (1) TK-negative mutants lacking TK expression and (2) TK-low-producer mutants have a lower expression of TK enzymatic activity, found in 95% of clinical ACV-resistant isolates, and (3) TK-altered mutants that contain substrate-specific mutations, found in 5% of clinical ACV^R^-HSV isolates [40]. Studies have proposed that TK mutants, in clinical isolates with a single G insertion into a 7-G string by +1 ribosomal frameshift during translation, can generate a low level of full-length active thymidine kinase [41,42].

Since the heterogeneous population of laboratory HSV strains contain TK variants, we wanted to examine the protein expression of TK following GFP-HSV-1 and ACV^R^-HSV-1 infection in the presence of ACV. The experiment stated above was repeated, and the viral inoculum was collected. Infected Vero cells were collected 20 h post-infection, and total cell lysate was subjected to western blot analysis using antibodies directed against HSV-1 infected cellular protein 0 (ICP0), glycoprotein D (gD), thymidine kinase (TK), and GAPDH (Figure 1E). For GFP-HSV-1 infection, we observed that the addition of 45 µM ACV substantially decreased the expression of ICP0 and TK, whereas the protein expression of gD was inhibited. In contrast, our western blot analyses demonstrated that for ACV^R^-HSV-1 infection, regardless of the addition of 45 µM ACV, ICP0 and gD expression was not altered. However, the expression of TK had a noticeable reduction with or without the addition of 45 µM ACV in both of the ACV^R^-HSV-1 viral stocks. Therefore, we propose that resistance in the ACV^R^ mutant is occurring through changes in the TK gene. Since we observed a considerable decrease in TK expression of ACV^R^-HSV-1 stock 2, we used this stock for subsequent experiments.

We investigated the inhibitory activity of 10 µM GA following ACV^R^-GFP-HSV-1 infection in Vero cell culture using plaque-forming assays (Figure 1F). In accordance with our previous results, an approximate 2-log difference in viral titer was seen between GFP-HSV-1 infected cells treated with 10 µM GA compared to vehicle-treated infected cells. In Figure 1F, GFP-HSV-1 infected cells treated with 10 µM ACV demonstrated an approximate 4-log decrease in viral titer than vehicle-treated cells. In comparison, ACV^R^-HSV-1 infected cells treated with 10 µM GA showed a 2-log reduction in viral titer compared to cells treated with DMSO and 10 µM ACV. Likewise, the images of the plaque assays demonstrate the qualitative effects of these results. These results demonstrate GA’s ability to inhibit ACV^R^- HSV-1, where ACV has no protective effect.

### 3.2. GA Formulated in HEC Gel Protects BALB/cJ Mice against GFP-HSV-1 Zosteriform Infection

The flank scarification model has been used to evaluate the efficacy of antiviral drugs and vaccines [37]. In a mouse flank model, HSV-1 is scratch inoculated onto the skin to expose the epidermal cells to the virus. The infection of the upper layers of the skin optimizes viral neuro-invasiveness, which closely resembles primary human HSV-1 infection. The virus spreads to the innervating sensory nerve endings, where it travels to the dorsal root ganglia (DRG) in the spinal cord. In reactivation, there is a retrograde spread of infection. HSV-1 travels from the spinal cord back to the skin, causing the development of zosteriform lesions along the dermatome of the nerve [37,43]. Previous studies have reported that BALB/cJ mice are more susceptible to HSV-1 due to the expression of *Rhs1^S^*, a Natural Killer (NK) complex-linked genetic locus, as assessed by the zosteriform skin infection model. *Rhs1* is responsible for rapid control of acute HSV-1 ganglionic infection and an increased frequency of latently infected neurons [44]. There is no difference in pathogenesis nor the establishment of latent HSV-1 infection in males and female mice; however, female BALB/cJ mice are commonly used for HSV epidermal scarification-zosteriform model [37].

We initially assessed if GA causes irritation or an allergic reaction, using a modified skin irritation model, based on a protocol previously described by Sekizawa et al. [34]. Our results indicated no visible signs of irritation in the application of 10 mM GA 24 h-to-120 h post-treatment (see Figure S2). Therefore, we continued to examine the ability of GA to protect against the early stage of cutaneous HSV-1 infection using the epidermal scarification-zosteriform model.

**Figure 2:**
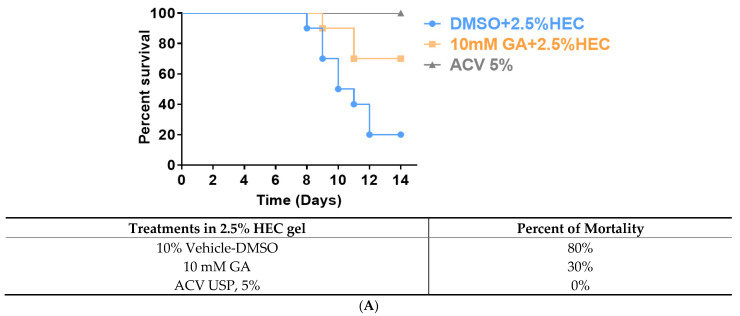

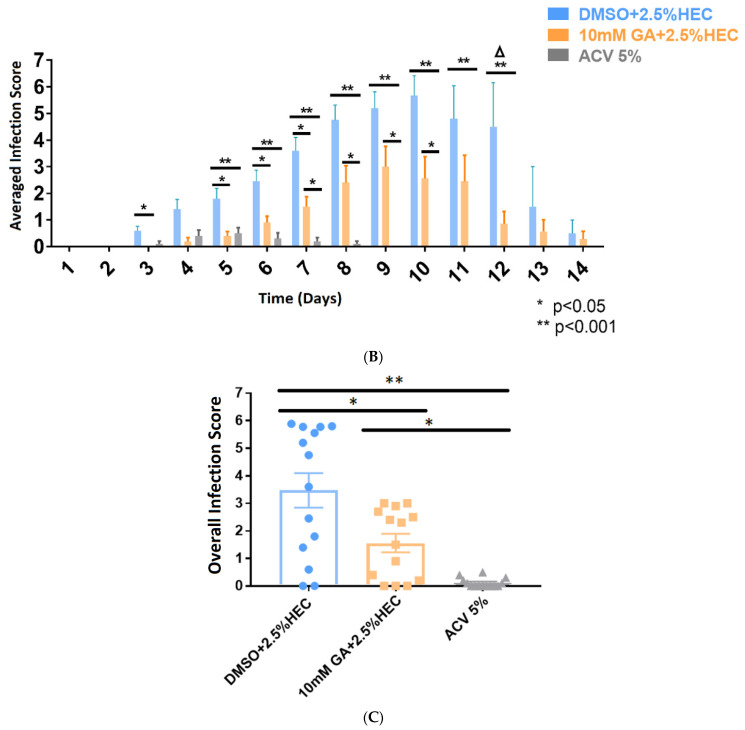
GFP-HSV-1 average zosteriform infection scores of BALB/cJ mice (N = 10/treatment), following the application of DMSO in 2.5% HydroxyEthyl Cellulose (HEC) gel, 10 mM GA in HEC gel, and ACV USP 5%. (**A**) Age-matched BALB/cJ mice were inoculated with 6 × 10^4^ PFU of GFP-HSV-1, given respective treatments, and monitored for survival for 14 days. (**B**) Averaged infection scores of surviving animals in each treatment group across 14 days. (**C**) Combined and averaged infection score of all animals per day for 14 days. GA-treated animals demonstrated a significant reduction in the appearance of vesicles and erosions compared to vehicle-treated control animals. (**B**) Student independent *t*-tests (2-tailed) and (**C**) One-way ANOVA [F(2, 39) = 12.31]; * *p* < 0.05, ** *p* < 0.001. Δ indicates that 8 out of 10 animals in the DMSO-treated group died by day 12 p.i. All error bars represent SEM.

We used 5–6 weeks old female BALB/cJ mice to test the efficacy of GA against zosteriform infection. Cutaneous GFP-HSV-1 infections were conducted using the epidermal scarification-zosteriform model, as previously described by Goel et al. [37]. For topical treatments, we formulated GA in 2.5% hydroxyethyl cellulose (HEC) gel. HEC is a gel that contains no active microbicide; it has been adopted as a placebo in many clinical trials of microbicides, and it has been included in studies where the active gel showed protection against HSV-2 acquisition [45,46]. All animals (N = 10/treatment, three treatments in total) were inoculated with 6.0 × 10^4^ PFU GFP-HSV-1-Strain 17+. The viral concentration was determined while establishing the laboratory’s zosteriform infection model (see Figure S3). The inoculation site was treated with 10% DMSO (vehicle-control) formulated in 2.5% HEC gel, 10 mM (equals to 10% in volume) GA in 2.5% HEC gel, or acyclovir (ACV) ointment, USP 5% (control for the standard of care). Each treatment was administered 1-to-2 h post-infection (p.i.) and continued twice daily, at 12-h intervals, for approximately 14 days. Animals were monitored p.i. twice daily for 14 days for any signs of deterioration. Signs of disease at the inoculation site were scored and recorded by the appearance of vesicles and erosions. Our results indicate that DMSO-treated animals had a 20% survival rate within 14 days (Figure 2A). GA-treated animals showed a 70% rate of survivability p.i., compared to the vehicle-treated control animals across 14 days (*p* < 0.001 indicated by Log-rank (Mantel-Cox) test; Figure 2A). The infection score of the surviving animals was averaged each day and analyzed to compare the effect of each treatment against HSV-1 infection. The DMSO-treated animals exhibited significantly increased severity of HSV-1 infection, characterized by the appearance of vesicles along the dermatome and severely compromised health (Figure 2B). Whereas, GA-treated animals demonstrated a significant reduction in the formation of vesicles and erosions compared to vehicle-treated control animals on days 3, 5, 6, and 7 (*p* < 0.001 and *p <* 0.05, indicated by multiple independent *t*-tests). As expected, we observed low infectivity scores and no mortality in animals treated with ACV USP, 5% (50 mg/gr); (Figure 2B). Analysis of infection scores averaged per day for each group indicated that treatment of 10 mM GA significantly reduces the severity and duration of infection over 14 days (one-way ANOVA; F(2, 39) = 12.31, *p* < 0.05) compared with the vehicle-treated controls for which the infection scores peaked around day 10 (Figure 2C).

### 3.3. GA Formulated in PEG Protects BALB/cJ Mice against GFP-HSV-1 Zosteriform Infection

Based on the significance of our previous results, we proposed to investigate the protective effects of GA formulated in a different vehicle for cutaneous application. We formulated GA in an FDA-approved vehicle containing polyethylene glycol (PEG), a penetration enhancer for topical dermatological treatments [47].

**Figure 3:**
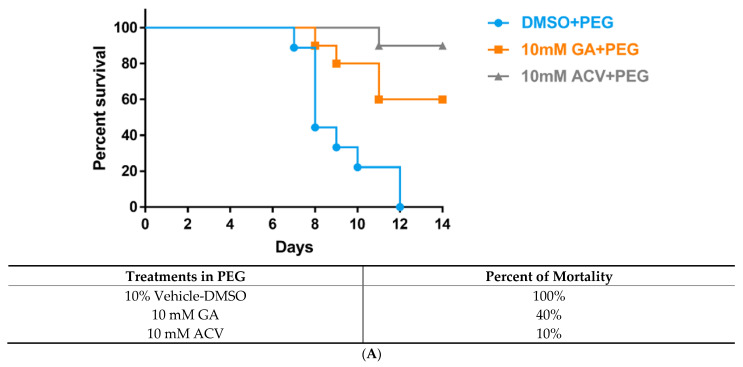

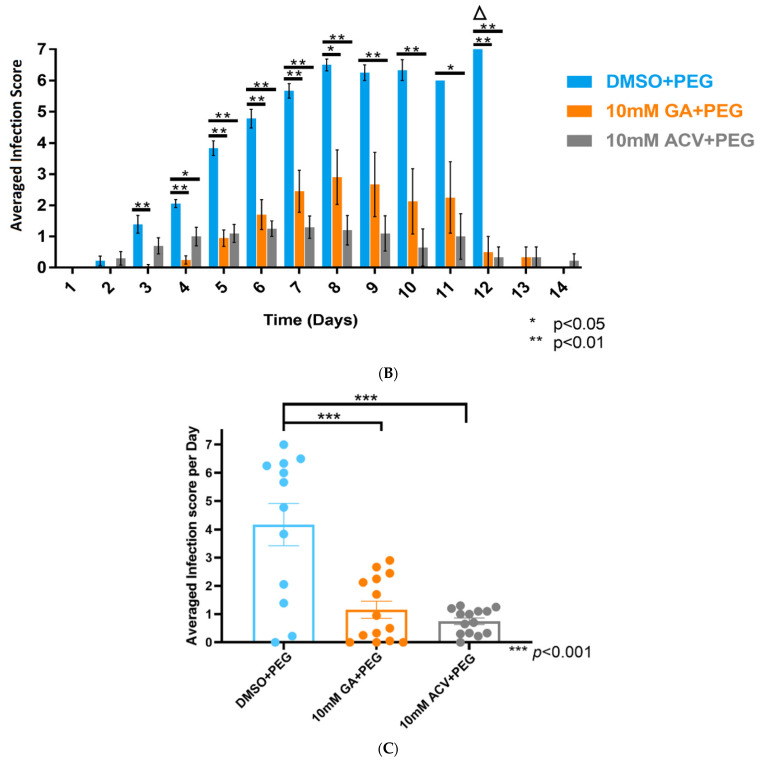
GFP-HSV-1 average zosteriform infection scores of BALB/cJ mice, following the application of 10% vehicle-DMSO in Polyethylene glycol (PEG) (N = 9/group), 10 mM GA in PEG (N = 10/group), and 10 mM Acyclovir in PEG (N = 10/group). (**A**) Age-matched BALB/cJ mice were inoculated with 6 × 10^4^ PFU of GFP-HSV-1, given respective treatments, and monitored for survival for 14 days. (**B**) Averaged infection scores of surviving animals in each treatment group across 14 days. (**C**) Combined infection score of all animals, in their respective groups, per day for 14 days. Student independent *t*-tests (2-tailed); * *p* < 0.05; ** *p* < 0.01; *** *p* < 0.001. Δ indicates that all DMSO-treated animals died on day 12. All error bars represent SEM.

Using the same methodology stated above, we treated infected BALB/cJ female mice (N = 10/treatment group) at the inoculation sites with either DMSO (vehicle-control) formulated in PEG, 10 mM GA in PEG, or 10 mM ACV in PEG. Compared to 0% survivability of animals treated with DMSO, GA-treated animals showed a 60% rate of survivability p.i. when compared across a 14-day study (*p* < 0.05 indicated by Log-rank (Mantel-Cox) test; Figure 3A). As expected, the ACV positive control protected 90% of the treated animals. The infection severity scores of the surviving animals were averaged each day and analyzed to compare the effect of treatments against HSV-1 infection. GA-treated animals demonstrated a significant reduction in the appearance of vesicles and erosions compared to vehicle-treated control animals from day 3, 4, 5, 6, 7, 8, and 12 (*p* < 0.01 and *p* < 0.05, indicated by multiple independent *t*-tests; Figure 3B). As expected, we observed low infectivity scores and a decreased mortality rate in animals treated with 10 mM ACV. Our results demonstrate a potent inhibition of HSV infection and faster healing following treatment with GA, which can be attributed to GA’s virucidal activity and its fusion inhibition ability preventing the cell-to-cell spread of infection. Based on our findings, infection scores for each group (averaged per day) demonstrate that the treatment of 10 mM GA significantly reduces the severity of infection over 14 days compared to the vehicle-treated control the infection scores peak around day 12 (Figure 3C).

### 3.4. GA Formulated in PEG Protects BALB/cJ Mice against ACV^R^-HSV-1 Zosteriform Infection

Due to the increasing emergence of antiviral resistant HSV, it was pertinent to test the protective effect of GA against resistant infections, for which ACV has no effect. Female BALB/cJ mice (N = 5/treatment, three treatments in total) were inoculated with 2.0 × 10^5^ PFU of ACV^R^-HSV-1.

Using the zosteriform model of TK-deficient HSV infection in BALB/c mice, studies have demonstrated that most isolates show impaired growth at the inoculation site compared with wild-type HSV virus [48]. TK-deficient isolates express a low level of TK functionality and have been shown to have low neurovirulence, whereas TK-negative mutants are unable to replicate in the nervous system. The majority of clinical mutants with low expression of TK have also shown little-to-no evidence of zosteriform spread following acute infection. Although these isolates have shown reduced pathogenicity, they can be reactivated sporadically from latently infected ganglia [44,48]. The in vivo viral concentration of ACV^R^-HSV-1 was determined while establishing the zosteriform infection model in our laboratory [48]. As expected, ACV^R^-HSV-1 did not form zosteriform infection, and no infected mice died. For our experiments, 2.0 × 10^5^ PFU of ACV^R^-HSV-1 was used to infect each mouse (N = 5/group). The infected site was treated with DMSO (vehicle-control) formulated in PEG, 10 mM GA in PEG, or 10 mM ACV in PEG. As stated in the methodology above, each treatment was administered 1-to-2 h p.i. and continued twice daily, at 12-h intervals, for 14 days. Each animal was housed separately, monitored twice daily for 14 days for any signs of deterioration. The signs of disease at the inoculation site were scored using the appearance of vesicles and or erosions.

**Figure 4:**
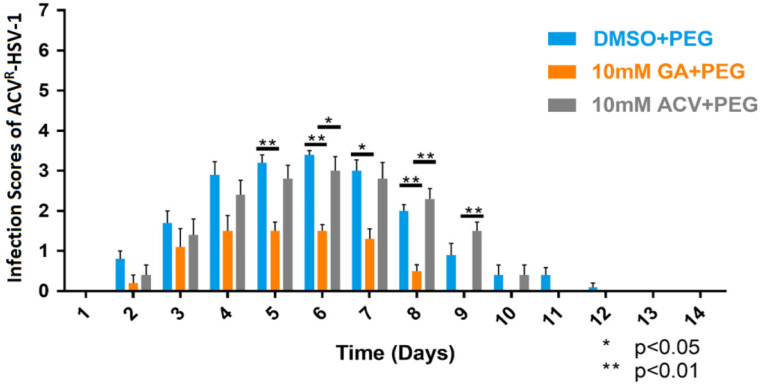
ACV^R^-HSV-1 average zosteriform infection scores of BALB/cJ mice, following the application of 10% DMSO in polyethylene glycol (PEG), 10 mM GA in PEG, and 10 mM ACV in PEG (N = 5/group). Age-matched BALB/cJ mice were inoculated with 2.0 × 10^5^ PFU of ACV^R^-HSV-1 using the epidermal scarification-zosteriform model. Each group received the respective treatments and were monitored for 14 days. The infection scores of surviving animals were averaged in each treatment group for each day. GA-treated animals demonstrated a significant reduction in the appearance of vesicles and erosions compared to vehicle-treated control animals. Student independent *t*-tests (2-tailed); * *p* < 0.05; ** *p* < 0.01. All error bars represent SEM.

Our results indicated 100% survivability of all animals, regardless of treatment. Animals treated with DMSO and ACV demonstrated similar severity of infection across 14 days of treatment. Whereas, GA-treated animals showed a significant reduction in the formation of vesicles and erosions compared to vehicle-treated control animals on days 5, 6, 7, and 8 (*p* < 0.01 and *p* < 0.05, indicated by multiple independent *t*-tests). Interestingly, GA-treated animals also demonstrated a significant reduction in the formation of vesicles and erosions compared to 10 mM ACV-treated animals on days 6, 8, and 9 (*p* < 0.01 and *p* < 0.05, indicated by multiple independent *t*-tests) (Figure 4). These results indicate that GA’s protective ability is effective against acyclovir-resistant HSV-1 cutaneous infection.

## 4. Discussion

Ginkgolic acid (GA) is a major constituent of *Ginkgo biloba* extract and has been used as an herbal supplement in Chinese medicine for centuries [18,19]. Recently, we discovered that GA inhibits herpes simplex virus type 1 (HSV-1) by inhibiting both fusion and viral protein synthesis. Our experiments suggest that GA inhibits virion entry by blocking the initial fusion event, as well as by disrupting the viral structure (virucidal activity) [31].

Here we show for the first time that GA formulated in two different vehicles inhibits HSV-1 cutaneous infection in a zosteriform model in BALB/cJ mice. GA significantly reduced mortality, the infection score, and the duration of the disease. Furthermore, we show for the first time that GA inhibited an acyclovir-resistant strain of HSV-1 in vitro, in vivo, and protected animals against ACV^R^-HSV-1 zosteriform infection.

Just as we and others have reported before, GA at higher concentrations has toxic effects [31,49,50]. To eliminate any toxicity or irritation when applied to the skin, we tested GA in an in vivo irritation test based on an irritation model developed by Sekizawa et al. [34]. BALB/cJ (N = 4/group) were treated with one dose of 10 mM GA or DMSO vehicle formulated in 2.5% HEC gel. All animals were monitored daily for signs of skin irritation/redness or infection following the OECD rating system [35,36]. Our results indicated no visible signs of irritation in all tested concentrations 24 h to 120 h post-treatment (Figure S2).

Hydroxyethylcellulose (HEC) is a gel that contains no active microbicide; it has been adopted as a placebo in many clinical trials of microbicides [45]. GA embedded in 2.5% HEC gel applied to the skin of HSV-1 infected BALB/cJ mice had a significant effect in protecting them compared to DMSO vehicle. The mortality in the DMSO vehicle-treated mice over 12 days was 80% compared to just 30% in the GA-treated mice (Figure 2A). Likewise, the infection scores were significantly lower in the GA-treated group, the latter also experiencing a faster healing process (Figure 2B,C).

Vehicles containing PEG, polyether compounds with repeating ethylene glycol units, are hydrophilic, especially in topical dermatological applications [47]. To evaluate the effectiveness of GA formulated in PEG applied to the skin of HSV-1 infected BALB/cJ mice we used 10% DMSO (vehicle-control) formulated in PEG, 10 mM GA in PEG, or 10 mM ACV in PEG. GA embedded in PEG gel had a significant effect in protecting HSV-1 infected BALB/cJ mice compared to the DMSO vehicle. The mortality in the DMSO vehicle-treated mice over 12 days was 100% compared to just 40% in the GA-treated mice (Figure 3A). Likewise, the infection scores were significantly lower in the GA-treated group, the latter also experiencing a faster healing process (Figure 3B,C).

Although improvements in treatments have dramatically reduced morbidity and mortality, ACV-resistance HSV strains have been isolated from immunocompromised patients with a prevalence of between 14% and 36% from stem cell transplant recipients [14]. Viral mutations resulting in ACV-resistance have been found in both TK UL23 (accounting for 95% of clinical isolates) and pol UL30 gene (5% of clinical isolates) [12,14]. Despite the prevalence of isolates, ACV^R^-HSV infection remains a critical problem for many patients, including those with HIV infection [4,17]. Thus, the emergence of resistant viral strains generates a genuine need to find and develop new anti-HSV agents.

Since GA’s mechanism of action includes both virucidal activity and fusion inhibition, GA was expected to keep its anti-HSV activity also against HSV-ACV^R^ strains. To test that, we generated HSV-ACV^R^ viruses and first evaluated GA’s effect on the latter in an in vitro assay (Figure 1F). While ACV treatment did not affect the titers of the HSV-ACV^R^ in vitro infection, GA significantly reduced the titer by approximately 2-log. These results confirmed GA’s ability to inhibit ACV^R^- HSV-1, where ACV has no protective effect.

In the light of our in vitro results of GA’s potency against ACV^R^-HSV-1 and our success in treating HSV-1 cutaneous infections, we expected that GA would also work in vivo against ACV^R^- HSV-1 cutaneous infection. While in vitro ACV^R^-HSV-1 infection is similar to wild-type HSV-1, studies have demonstrated that when using the zosteriform model of TK-deficient HSV infection in BALB/c mice, the majority of isolates show impaired growth at the inoculation site when compared with wild-type HSV virus [48]. TK-deficient isolates express a low level of TK functionality and have been shown to have low neurovirulence, whereas TK-negative mutants are unable to replicate in the nervous system. The majority of the clinical mutants with low expression of TK have also shown little to no evidence of zosteriform spread following acute infections. Although these isolates have shown reduced pathogenicity, they can be reactivated sporadically from latently infected ganglia [44,48]. For our experiments, 2.0 × 10^5^ PFU of ACV^R^-HSV-1 were used to infect each mouse, and as expected, the ACV^R^-HSV-1 did not form zosteriform infection, and no mortality was observed post-infection. However, GA-treated animals demonstrated a significant reduction in the formation of vesicles and erosions, with a faster healing process compared to vehicle-treated and ACV-treated animals (Figure 4). These results demonstrate that GA is effective against acyclovir-resistant HSV-1 cutaneous infection.

While our results are significant compared to placebo in the wild-type HSV-1 infected mice, total resolution of infection was not achieved. Possible reasons for that are insufficient delivery of GA to the skin due the vehicles used (current topical commercial formulations use more elaborated formulas containing liposomes, transferosomes, solid lipid nanoparticles, cyclodextrins, or sol-gel microcapsules) [51] or of the decreased number of applications (the recommended frequency of acyclovir cream for the treatment of HSV infection is a minimum of five times daily).

In summary, here we show for the first time that GA is an efficient anti-HSV agent for the treatment of cutaneous infection. Furthermore, we show that GA is effective against HSV-ACV^R^ infection when ACV treatment has no effect. Since GA’s mechanism of action includes virucidal activity and fusion inhibition, it is expected to work alone or synergistically with other anti-HSV drugs such as nucleoside analogues. Finally, as we previously reported, GA has a broad spectrum of fusion inhibition of all three classes of fusion proteins, and therefore we anticipate it to be effective against cutaneous and other forms of viral infections.

## Data Availability

The data presented in this study are available upon request from the corresponding author.

## Supplementary Materials

The following are available online at https://www.mdpi.com/1999-4915/13/1/86/s1, Figure S1: Signs of disease at the inoculation site for scoring by the appearance of vesicles and erosions, Figure S2: Skin Irritation test, Figure S3: Verification of Zosteriform HSV-1 infection model in-vivo (N = 5/vial dose).
