# Supplementary material for: Ginkgolic Acid Inhibits Herpes Simplex Virus Type 1 Skin Infection and Prevents Zosteriform Spread in Mice
Source: Viruses. 2021 Jan 9;13(1):86. doi: 10.3390/v13010086 (PMC7826900; doi:10.3390/v13010086)
Supplement: Supplementary file 1 [file viruses-13-00086-s001.pdf]

Supplementary Information

Ginkgolic Acid Inhibits Herpes Simplex Virus Type 1 Skin Infection and Prevents Zosteriform Spread in Mice

Maimoona S. Bhutta [1], Oren Shechter [1], Elisa S. Gallo [2], Stephen D. Martin [1], Esther Jones [1], Gustavo F. Doncel [3,4] and Ronen Borenstein [1,*]

[1] Department of Microbiology and Molecular Cell Biology, Eastern Virginia Medical School, Norfolk, VA 23507, USA; bhuttam@evms.edu (M.S.B.); orenshechter@yahoo.com (O.S.); MartinS1@EVMS.EDU (S.D.M.); JonesEB@EVMS.EDU (E.J.)

[2] Board-Certified Dermatologist and Independent Researcher, Norfolk, VA 23507, USA;

[3] CONRAD, Arlington, VA 22209, USA; DoncelGF@EVMS.EDU

[4] Department of Obstetrics and Gynecology, Eastern Virginia Medical School, Norfolk, VA 23507, USA

* Correspondence: BorensR@evms.edu

Infection Score	GRADE
No lesions	0
Local site lesions	1-2
Distant site zosteriform lesions along the dermatome	3–5
Progression to severely compromised health	6
Death	7

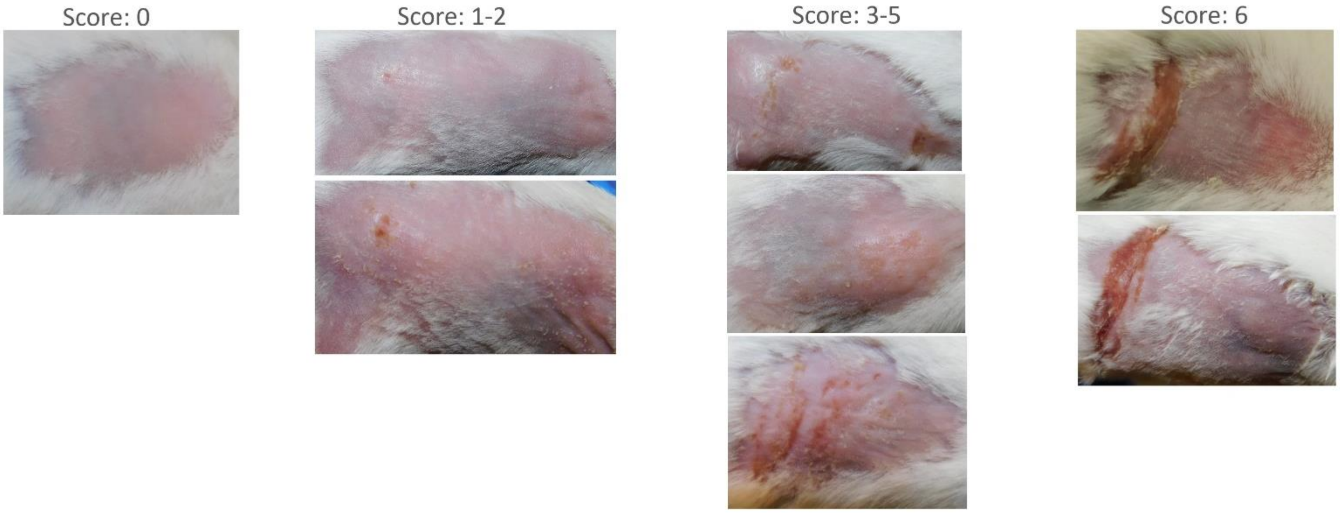

Figure S1. Signs of disease at the inoculation site for scoring by the appearance of vesicles and erosions. Outcomes were graded as outlined in the infection score chart. The images represent the respective infection scores on the right flank of BALB/cJ mice. The infection score increases down the column of each score range, e.g. for the score of 1-2, the top image represents an infectivity score of 1, and the bottom image represents an infectivity score of 2.

GA does not irritate the skin of BALB/cJ mice

Female BALB/cJ (4-5 weeks of age) mice were purchased from Jackson Laboratories (JAX stock #000651). To assess whether the dermal application of GA causes irritation or an allergic reaction, we used the skin irritation protocol, as previously described by Sekizawa et al. [34]. Under isoflurane gas anesthesia, the hair on the right flank of BALB/cJ (N=4/group) was shaved (1 x 2 cm area) and depilated with Nair. 10mM GA and DMSO vehicle were formulated in 2.5% HEC gel and applied on shaven skin using a sterile plastic end of a cotton swab. Mice were caged separately to prevent licking of gel solutions. All animals were monitored daily for signs of skin irritation/redness or infection in accordance with the Organization for Economic Co-operation and Development (OECD) rating system [35,36].
Our results indicated no visible signs of irritation in all tested concentrations 24 hours-to-120 hours post-treatment (Figure S2). We determined that the application of GA does not irritate the skin; therefore, we continued to examine GA's protective effect against the early stage of cutaneous HSV-1 infection using the epidermal scarification-zosteriform model.

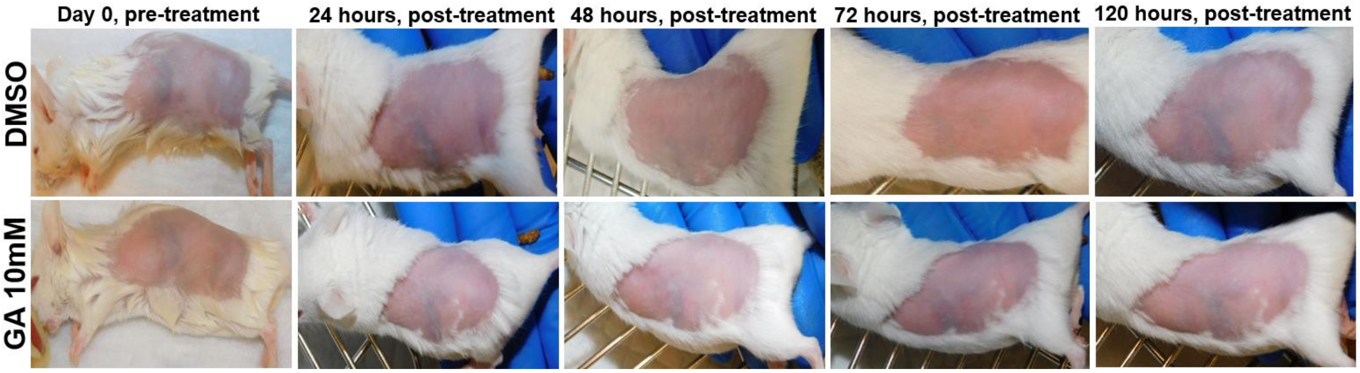

Figure S2. Skin Irritation test. Representative images of mice (N=4) from two treatment groups. Female BALB/cJ treated with 10% DMSO-HEC (10% DMSO embedded in HEC gel) vs. 10% GA-HEC (10mM GA (10% in volume), embedded in HEC gel).

Validation of HSV Zosteriform Infection Model

Cutaneous GFP-HSV-1 infections were conducted using the epidermal scarification-zosteriform model, as previously described by Goel et al. [37]. Female BALB/cJ mice (N=5/group, 2 groups total) were inoculated with GFP-HSV1-Strain 17+ concentration of 6 x 10^4 PFU or 6 x 10^5 PFU, respectively. Animals were monitored twice daily post-infection (p.i.) for 14 days for any signs of deterioration. Signs of disease at the inoculation site were scored and recorded by the appearance of vesicles and erosions (Figure S1). Our results indicated that at the highest dose, 100% of mice died within 14 days, with a median survival time of 8 days (Figure S3A). The lower dose of 6 × 10^4 PFU caused only 60% lethality with a median survival time of 9 days. We observed the early onset of infected vesicles in both groups from day 3 to day 5 p.i., respectively, whereas the infectivity peaked around day 9 (Figure S3B). It is important to note that animals infected with a dose of 6 × 10^4 PFU demonstrated signs of healing 11 days p.i (Figure S3C), which renders the lower dose suitable for comparing the efficacy of our treatments in BALB/cJ mice.

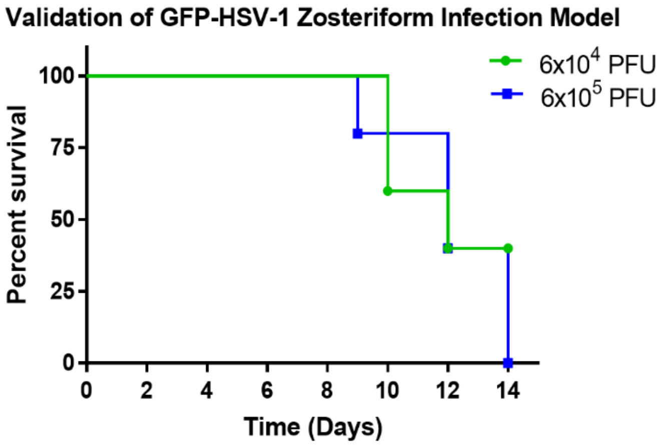

Viral Dosage	Percent Mortality
6 X 10^4 PFU	100%
6 X 10^5 PFU	60%

(A)

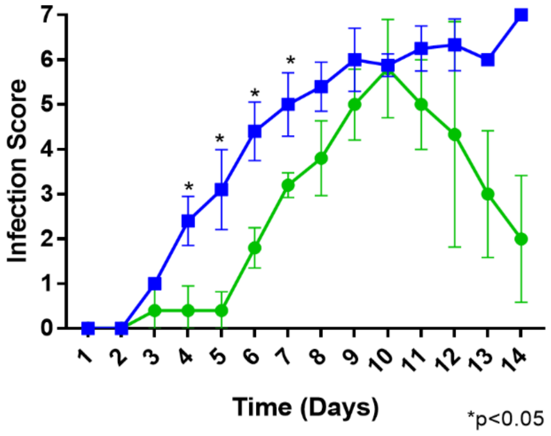

(B)

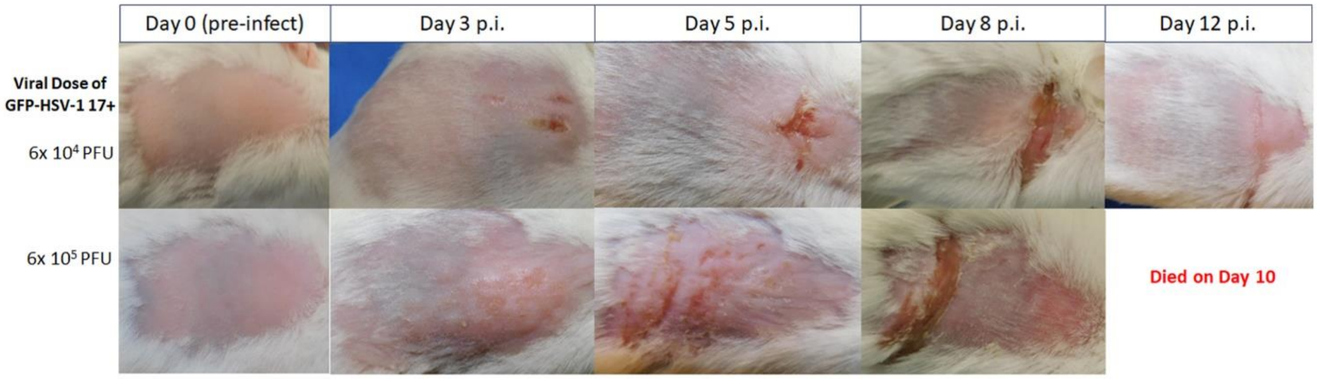

(C)

Figure S3. Verification of Zosteriform HSV-1 infection model *in-vivo* (N=5/vial dose). (A) Age-matched BALB/cJ mice were inoculated with 6 x 10^4 PFU or 6 x 10^5 PFU of GFP-HSV1-Strain 17+ and monitored for survival for 14 days. (B) Mice inoculated with a viral dose of 6 x 10^5 PFU demonstrated a significantly greater appearance of vesicles and erosions than animals inoculated with 6 x 10^4 PFU. (C) Representative images of female BALB/cJ mice in an epidermal scarification-zosteriform model. Student independent t-tests (2-tailed). * $p<0.05$. All error bars represent SEM.
